# Evaluating the Use of Amoxicillin in Community-Acquired Pneumonia Patients With Low Confusion, Urea, Respiratory Rate, Blood Pressure, and Age ≥65 Years (CURB-65) Scores: A Retrospective Study

**DOI:** 10.7759/cureus.86477

**Published:** 2025-06-21

**Authors:** Trimanjot Singh Anand, Wilsonne Andrew Kyle T Chua, Soo Hyun Jung, Daniela Cristea-Nicoara

**Affiliations:** 1 Internal Medicine, University Hospitals of Leicester NHS Trust, Leicester, GBR; 2 Respiratory Medicine, University Hospitals of Leicester NHS Trust, Leicester, GBR

**Keywords:** amoxicillin, amoxicillin therapy, antibiotic stewardship, community-acquired pneumonia, curb-65 score, empirical antibiotic therapy, physician guideline adherence

## Abstract

Community-acquired pneumonia (CAP) remains a significant cause of morbidity and mortality worldwide, necessitating precise antibiotic strategies guided by severity assessment tools such as the confusion, urea, respiratory rate, blood pressure, and age ≥65 years (CURB-65) score. This retrospective study evaluated initial antibiotic choices for patients with low CURB-65 scores (0 and 1) at Glenfield Hospital, University Hospitals of Leicester NHS Trust, focusing on the role of amoxicillin in early-stage pneumonia management. Data from 400 patients across various hospital settings in 2023 were analyzed. Co-amoxiclav was the most prescribed antibiotic, used in 226 cases (62.78%), while amoxicillin was prescribed in 46 cases (12.78%). Despite the small sample size and lack of comorbidity adjustment, the study found a high discharge rate (40 patients, or 86.95%) and low readmission rate (four patients, or 8.70%) for patients initially treated with amoxicillin, supporting its potential as a first-line therapy for low-risk CAP. However, broader studies with more comprehensive data are needed to confirm these findings and refine clinical guidelines, including biomarker integration for more precise prescribing.

## Introduction

Community-acquired pneumonia (CAP) is a leading cause of hospital admissions and mortality worldwide, with significant clinical and economic impacts [1]. The British Thoracic Society (BTS) and other major health bodies emphasize using validated severity assessment tools, such as the confusion, urea, respiratory rate, blood pressure, and age ≥65 years (CURB-65) score, to guide antibiotic selection [1-3]. However, despite these guidelines, antibiotic prescribing practices vary widely, often influenced by clinician experience, patient comorbidities, and institutional protocols [4].

Amoxicillin, a narrow-spectrum beta-lactam antibiotic, remains a first-line option for low-severity CAP due to its targeted activity against common respiratory pathogens and lower risk of promoting antimicrobial resistance [5]. In contrast, broader-spectrum agents like co-amoxiclav, meropenem, and piperacillin/tazobactam are associated with higher risks of adverse effects, including *Clostridium difficile* infection and microbiome disruption [6,7]. Given the growing focus on antibiotic stewardship, it is essential to evaluate whether initial amoxicillin therapy can effectively manage low-risk CAP without compromising patient outcomes.

This study aimed to assess the real-world impact of amoxicillin as an initial therapy for patients with low CURB-65 scores, providing critical insights for optimizing antibiotic selection and enhancing guideline adherence.

This study was also presented as a poster during the National Foundation Doctors Presentation Day on 17 January 2025 at Sheffield, United Kingdom.

## Materials and methods

This retrospective study included 400 patients with CURB-65 scores of 0 to 1 who presented across the Clinical Decisions Unit (CDU), Same Day Emergency Care (SDEC), Emergency Department (ED), General Practice (GP), and Outpatient Clinics (OPD) at Glenfield Hospital in 2023. Inclusion criteria were kept broad regardless of age, co-morbidities, or other risk factors. This approach aimed to demonstrate a diverse and representative sample of low-severity CAP cases in Leicester.

Patient data were identified and extracted using the hospital’s electronic patient record system, NerveCentre (Nervecentre Software Ltd., Nottingham, UK). CURB-65 scores were recorded at the time of presentation. The study focused on two primary outcomes: the initial antibiotic prescribed and the readmission rate. Antibiotic selection was assessed through a review of prescribing records documented at admission. Readmission was defined as any unplanned return to hospital or clinical escalation identified during the six- to eight-week follow-up period with the Specialist Pneumonia Intervention Nursing (SPIN) team. Readmission rates were calculated per antibiotic group, based on the total number of patients initially treated with each respective agent.

The CURB-65 score was used in accordance with published clinical guidelines for both quality improvement and research purposes. Permission was not required as the score is a publicly available, non-proprietary clinical tool, and no commercial software was used in its calculation.

All data were anonymized prior to analysis, and the dataset was handled using secure, encrypted hospital systems. Descriptive statistical analysis was performed using Microsoft Excel (Microsoft® Corp., Redmond, WA, USA), reporting outcome frequencies and proportions across the cohort. No inferential statistical testing was applied, given the exploratory and observational nature of the study.

All personal identifiers were removed to protect patient privacy, and data were accessed through secure, encrypted hospital systems. In accordance with institutional guidelines and local data protection regulations, formal ethics approval was waived. This study was conducted retrospectively, and patient consent was not required due to the fully anonymized nature of the data, ensuring compliance with relevant data protection standards.

## Results

Table 1 summarizes the initial antibiotic prescribing patterns for the study cohort. Co-amoxiclav was the most prescribed antibiotic (62.78%), followed by amoxicillin (12.78%), doxycycline (9.72%), and various broad-spectrum agents, including meropenem (5.28%) and piperacillin/tazobactam (1.39%). Approximately 10% of patients had incomplete or unavailable antibiotic data.

**Table 1:** Raw data on initial antibiotic therapy received by patients

Antibiotic	Number of patients	Percentage (%)
Co-amoxiclav	226	62.78
Amoxicillin	46	12.78
Doxycycline	35	9.72
Meropenem	19	5.28
Clarithromycin	19	5.28
Piperacillin + tazobactam	6	1.39
Levofloxacin	4	1.11
Ciprofloxacin	4	1.11
Azithromycin	2	0.50
No data	40	10.00

Table 2 focuses on discharge and readmission outcomes for the two most frequently prescribed antibiotics: amoxicillin and co-amoxiclav. Among patients initially treated with amoxicillin, 40 were discharged (86.95%), and four were readmitted (8.70%). Of the readmitted patients, two (50%) required escalation to co-amoxiclav, while one (25%) received a second course of amoxicillin followed by doxycycline.

**Table 2:** Data on number of discharged and readmitted patients primarily treated with amoxicillin and co-amoxiclav

Antibiotics	Discharged patients	Readmitted patients
Amoxicillin	40	4
Co-amoxiclav	408	28

In comparison, 408 patients (93.58%) treated with co-amoxiclav were discharged, and 28 (6.42%) were readmitted. While co-amoxiclav showed a slightly higher discharge rate and lower readmission rate, this must be interpreted with caution due to the substantial difference in sample sizes between the two groups.

Overall, these findings suggest that amoxicillin may be a clinically effective first-line option for patients with low-risk CAP (CURB-65 score 0 and 1). However, the disparity in sample sizes and potential confounding factors limits the strength of this conclusion. Further studies with larger and more balanced treatment groups are recommended to confirm these preliminary observations.

## Discussion

The findings of this study reinforce the potential of amoxicillin as an effective first-line therapy for low-risk CAP, particularly in patients with CURB-65 scores of 0 and 1. Amoxicillin remains a cornerstone of initial CAP management due to its narrow-spectrum activity, excellent oral bioavailability, and proven efficacy against common respiratory pathogens like *Streptococcus pneumoniae*, *Haemophilus influenzae*, and *Moraxella catarrhalis* [1,2]. Importantly, amoxicillin is associated with a lower risk of adverse effects, including *C. difficile *infection and disruption of the gut microbiome, compared to broader-spectrum agents like co-amoxiclav [4,5]. The high discharge rate (86.95%) and relatively low readmission rate (8.70%) observed in this study support the continued use of amoxicillin as an effective, guideline-concordant choice for patients with low-severity CAP. This aligns with BTS and American Thoracic Society (ATS) guidelines, which recommend amoxicillin as the preferred first-line option in the absence of risk factors for atypical pathogens or severe disease [1,6]. However, the absence of microbiological data in this study limits the ability to confirm that amoxicillin was appropriately targeted against these pathogens, highlighting the need for future studies incorporating microbiological testing to better define its role in CAP management [7].

Despite the established benefits of amoxicillin, this study found that co-amoxiclav was the most prescribed antibiotic, accounting for 62.78% of initial therapies. This preference likely reflects clinician concerns about potential beta-lactamase-producing pathogens, broader Gram-negative coverage, and empirical coverage for mixed infections, which amoxicillin alone may not address [8]. Co-amoxiclav combines amoxicillin with the beta-lactamase inhibitor clavulanic acid, extending its spectrum to include beta-lactamase-producing strains of *H. influenzae*, *M. catarrhalis*, and certain *Enterobacteriaceae* [9]. However, this broader coverage comes at a cost, including increased risks of *C. difficile *infection, adverse drug reactions, and disruption of the gut microbiome [4,5]. The overuse of broad-spectrum antibiotics like co-amoxiclav, despite low CURB-65 scores, highlights a potential gap in adherence to established prescribing guidelines and underscores the need for more precise, pathogen-targeted therapy. This over-reliance on co-amoxiclav, despite its broader spectrum, could contribute to the rising threat of antimicrobial resistance and warrant ongoing stewardship efforts to optimize initial antibiotic choices [7,8].

Emphasis should also be given to introducing biomarkers into clinical guidelines to improve antibiotic precision, reducing the need for broad-spectrum agents and minimizing resistance risk [7,10]. Biomarkers can help differentiate bacteria from viral infections, guide initial antibiotic selection, and reduce unnecessary antibiotic use. For example, procalcitonin (PCT), a peptide released in response to bacterial infections, has been shown to reduce antibiotic exposure by up to 40% in lower respiratory tract infections without compromising patient outcomes [11,12]. A landmark trial, the ProHOSP study, demonstrated that PCT-guided algorithms significantly reduced the duration of antibiotic therapy and the frequency of broad-spectrum antibiotic use in patients with CAP, aligning closely with the goals of antimicrobial stewardship [13].

C-reactive protein (CRP) is another widely studied biomarker that can aid in the early identification of bacterial infections. Point-of-care CRP testing has been successfully implemented in primary care settings, providing rapid diagnostic support for antibiotic decisions. CRP levels have been shown to correlate with the severity of bacterial infections, helping to distinguish bacterial pneumonia from viral pneumonia and guiding the decision to initiate or withhold antibiotics [10]. For instance, a recent meta-analysis found that CRP-guided antibiotic therapy in respiratory infections reduced unnecessary antibiotic prescriptions by 22-36% without increasing the risk of treatment failure [14].

Beyond individual biomarkers, composite diagnostic scores that incorporate biomarkers alongside traditional clinical parameters offer promising improvements in pneumonia management. For example, the Pro-Adrenomedullin (ProADM) score, which combines adrenomedullin levels with traditional severity scores like CURB-65, has been shown to improve early risk stratification and reduce hospitalizations without compromising patient safety [15]. Similarly, the SMART-COP score, which incorporates CRP and other physiological markers, has demonstrated superior predictive accuracy for severe CAP requiring intensive care compared to CURB-65 alone [16]. Integrating such tools into routine practice could enhance the precision of antibiotic prescribing, further supporting antimicrobial stewardship efforts. However, challenges remain in integrating these biomarkers into routine practice. Cost, availability, and the need for clinician training are significant barriers, as is the risk of over-reliance on biomarker results at the expense of clinical judgment [7]. Despite these limitations, the potential for biomarkers to improve antibiotic stewardship and reduce antimicrobial resistance makes them a promising area for future research and clinical application. In this context, the CURB-65 score was selected for this study due to its widespread use, simplicity, and endorsement by both the BTS and the National Institute for Health and Care Excellence (NICE) guidelines. Unlike more comprehensive scoring systems such as the Pneumonia Severity Index (PSI) or SMART-COP, CURB-65 relies on readily available clinical and laboratory parameters, making it practical for routine clinical use and consistent documentation. This ease of use also makes it particularly well-suited for retrospective data extraction in real-world NHS settings.

This study also has multiple limitations. Firstly, it did not include detailed comorbidity data, which is a significant factor in CAP outcomes. Patients with chronic conditions such as chronic obstructive pulmonary disease (COPD), diabetes, or heart failure often have more severe disease presentations and may respond differently to initial antibiotic therapy. The absence of comorbidity data limits the ability to fully assess the appropriateness of amoxicillin as a first-line agent in these higher-risk populations.

Secondly, the study lacks microbiological confirmation of pneumonia diagnoses. While the CURB-65 score is a widely accepted clinical tool, it does not account for the specific pathogen responsible for the infection. This omission prevents precise evaluation of amoxicillin’s effectiveness against common CAP pathogens like *S. pneumoniae* and atypical organisms such as *Mycoplasma pneumoniae* and *Legionella pneumophila*. Without microbiological data, it is difficult to determine whether amoxicillin was appropriately chosen based on the causative agent, potentially leading to overestimation of its efficacy.

Finally, the study was retrospective and relied on data extracted from electronic health records, which can introduce selection bias and data entry errors. Approximately 10% of patients had incomplete or unavailable antibiotic data, further limiting the strength of the findings. Future studies should aim to include comprehensive comorbidity and microbiological data to provide a more accurate assessment of amoxicillin’s role in CAP management.

## Conclusions

This study reinforces the potential role of amoxicillin as an effective first-line therapy for patients with low-risk CAP based on its high discharge rates and low readmission rates. These findings underscore the importance of adhering to guideline-recommended antibiotic choices, particularly in an era where antimicrobial resistance poses a significant global health threat. By selecting narrow-spectrum antibiotics like amoxicillin when clinically appropriate, clinicians can reduce the unintended consequences of broad-spectrum overuse, such as *C. difficile* infections, disruption of gut microbiota, and the acceleration of resistance. However, the retrospective design, absence of comorbidity data, and limited sample size of this study highlight the need for larger, prospective studies with integrated biomarker guidance, such as CRP and PCT, to better define the clinical scenarios where amoxicillin remains the optimal choice. Future research should focus on developing precise, patient-centered prescribing algorithms that integrate rapid diagnostics and personalized risk assessment, ensuring that each antibiotic choice is both evidence-based and context-specific.

Ultimately, this study contributes valuable real-world data to the ongoing conversation around antibiotic stewardship, providing a foundation for more precise and responsible prescribing practices in CAP management. With broader application, such targeted approaches can significantly reduce healthcare costs, improve patient outcomes, and slow the spread of antibiotic resistance.

## References

[1] Lim WS, Baudouin SV, George RC (2009). BTS guidelines for the management of community acquired pneumonia in adults: update 2009. Thorax.

[2] Metlay JP, Waterer GW, Long AC (2019). Diagnosis and treatment of adults with community-acquired pneumonia. An official clinical practice guideline of the American Thoracic Society and Infectious Diseases Society of America. Am J Respir Crit Care Med.

[3] Lim WS, van der Eerden MM, Laing R (2003). Defining community acquired pneumonia severity on presentation to hospital: an international derivation and validation study. Thorax.

[4] Chalmers JD, Akram AR, Singanayagam A, Wilcox MH, Hill AT (2016). Risk factors for Clostridium difficile infection in hospitalized patients with community-acquired pneumonia. J Infect.

[5] Brown KA, Langford B, Schwartz KL, Diong C, Garber G, Daneman N (2021). Antibiotic prescribing choices and their comparative C. difficile infection risk: a longitudinal case-cohort study. Clin Infect Dis.

[6] Mandell LA, Wunderink RG, Anzueto A (2007). Infectious Diseases Society of America/American Thoracic Society consensus guidelines on the management of community-acquired pneumonia in adults. Clin Infect Dis.

[7] Seok H, Park DW (2024). Role of biomarkers in antimicrobial stewardship: physicians' perspectives. Korean J Intern Med.

[8] Livermore DM, Hope R, Reynolds R, Blackburn R, Johnson AP, Woodford N (2013). Declining cephalosporin and fluoroquinolone non-susceptibility among bloodstream Enterobacteriaceae from the UK: links to prescribing change?. J Antimicrob Chemother.

[9] Kaye KS, Pogue JM (2015). Infections caused by resistant Gram-negative bacteria: epidemiology and management. Pharmacotherapy.

[10] Van Hecke O, Bjerrum L, Gentile I (2023). Guidance on C-reactive protein point-of-care testing and complementary strategies to improve antibiotic prescribing for adults with lower respiratory tract infections in primary care. Front Med (Lausanne).

[11] Schuetz P, Wirz Y, Mueller B (2018). Procalcitonin testing to guide antibiotic therapy in acute upper and lower respiratory tract infections. JAMA.

[12] Christ-Crain M, Jaccard-Stolz D, Bingisser R, Gencay MM, Huber PR, Tamm M, Müller B (2004). Effect of procalcitonin-guided treatment on antibiotic use and outcome in lower respiratory tract infections: cluster-randomised, single-blinded intervention trial. Lancet.

[13] Schuetz P, Christ-Crain M, Thomann R (2009). Effect of procalcitonin-based guidelines vs standard guidelines on antibiotic use in lower respiratory tract infections: the ProHOSP randomized controlled trial. JAMA.

[14] Aabenhus R, Jensen JU, Jørgensen KJ, Hróbjartsson A, Bjerrum L (2014). Biomarkers as point-of-care tests to guide prescription of antibiotics in patients with acute respiratory infections in primary care. Cochrane Database Syst Rev.

[15] Christ-Crain M, Morgenthaler NG, Stolz D (2006). Pro-adrenomedullin to predict severity and outcome in community-acquired pneumonia [ISRCTN04176397]. Crit Care.

[16] Charles PG, Wolfe R, Whitby M (2008). SMART-COP: a tool for predicting the need for intensive respiratory or vasopressor support in community-acquired pneumonia. Clin Infect Dis.

